# Joint moments during sprinting in unilateral transfemoral amputees wearing running-specific prostheses

**DOI:** 10.1242/bio.039206

**Published:** 2019-01-23

**Authors:** Yuta Namiki, Satoru Hashizume, Akihiko Murai, Yoshiyuki Kobayashi, Hiroshi Takemura, Hiroaki Hobara

**Affiliations:** 1 Artificial Intelligence Research Center, National Institute of Advanced industrial Science and Technology, 135-0064 Tokyo, Japan; 2 Graduate School of Science and Technology, Tokyo University of Science, 278-8510 Tokyo, Japan; 3 Human Informatics Research Institute, National Institute of Advanced industrial Science and Technology, 135-0064 Tokyo, Japan; 4 Faculty of Science and Technology, Tokyo University of Science, 278-8510 Tokyo, Japan

**Keywords:** Amputees, Locomotion, Prosthetic sprinting, Paralympics

## Abstract

Knowledge of joint moments will provide greater insight into the manner in which lower-extremity amputees wearing running-specific prostheses regain running capacity and compensate for replacement of an active leg with a passive prosthetic implement. Thus, the purpose of this study was to investigate three-dimensional joint moments during sprinting for unilateral transfemoral amputees wearing running-specific prostheses. Ten sprinters with unilateral transfemoral amputation performed maximal sprinting at the 22 m mark while wearing running-specific prostheses. Joint moments were calculated through an inverse dynamics approach. All peak flexion and extension moments in the prosthetic leg were found to be lower than those of the intact leg, except for the peak plantar flexion moment. In the frontal plane, the peak adduction and abduction moments in the prosthetic leg were generally lower than those of the intact leg. The peak internal rotation moments differed significantly between the legs, but the peak external rotation moments did not. The results of the present study suggest that asymmetric joint moment adaptations occur for unilateral transfemoral amputees to compensate for replacement of the biological leg with a passive prosthetic knee joint and running-specific prosthesis.

## Abstract (precis)

**Summary:** Asymmetric joint moment adaptations occur for unilateral transfemoral amputees to compensate for replacement of the biological leg with a passive prosthetic knee joint and running-specific prosthesis.

## INTRODUCTION

Carbon-fiber running-specific prostheses (RSPs) allow individuals with lower-extremity amputation to regain running capacity, partly by providing the residual leg with a spring-like function. Understanding of both the running mechanics employed by individuals with lower-extremity amputation and the biomechanical functions of the prostheses during running contributes to further development of rehabilitation techniques and prosthesis design (Hobara et al., 2013). Further, knowledge of the running mechanics will provide greater insight into the manner in which lower-extremity amputees wearing RSPs regain running capacity and compensate for replacement of an active leg with a passive prosthetic implement.

Several studies reported that athletes with transtibial amputation wearing RSPs have asymmetrical vertical ground reaction force (GRF) and joint kinetics between prosthetic (PST) and intact (INT) legs across a wide range of speeds from 3.0 m/s up to top speed (Beck et al., 2017; Buckley, 2000; Grabowski et al., 2010; McGowan et al., 2012; Willwacher et al., 2017). For transfemoral amputees, a previous study demonstrated that unilateral transfemoral amputees wearing RSPs exhibited smaller vertical and braking components of GRFs for the PST than the INT during maximal sprinting (Makimoto et al., 2017). In addition, a recent study demonstrated that leg stiffness in the PST was approximately 12% smaller than that of the INT in unilateral transfemoral amputees wearing RSP during maximal sprinting (Sano et al., 2017). However, no study has reported joint kinetics during sprinting in unilateral transfemoral amputees.

Joint moment is a commonly-used biomechanical parameter to enhance our understanding of muscular effort and joint control during running. It is known that unilateral transtibial amputees with RSPs exhibit asymmetric joint moment outputs between the PST and INT during running (Baum et al., 2018; Buckley, 2000), however, it remains unclear if differences between the PST and INT truly exist for other amputation levels, such as those sustained by unilateral transfemoral amputees. Indeed, Buckley (1999) showed that the prosthetic knees of unilateral transfemoral amputees were fully extended during the stance phase, yielding an asymmetric sprinting strategy between the PST and INT to compensate for the lack of biological knee and ankle function. However, although the running mechanics of unilateral transfemoral amputees was partly investigated in the above mentioned studies, little is known about joint moments for this population.

The purpose of the present study was to investigate joint moments during sprinting for unilateral transfemoral amputees wearing RSPs. Sprinters required to generate joint moment to bear the external moment due to GRF during stance phase. According to a previous study (Makimoto et al., 2017), unilateral transfemoral amputees during sprinting exhibited significantly smaller GRFs in the PST than in the INT. Therefore, we hypothesized that the joint moments of the PST would be smaller than those of the INT in unilateral transfemoral amputees during sprinting.

## RESULTS

**Fig. 1.**
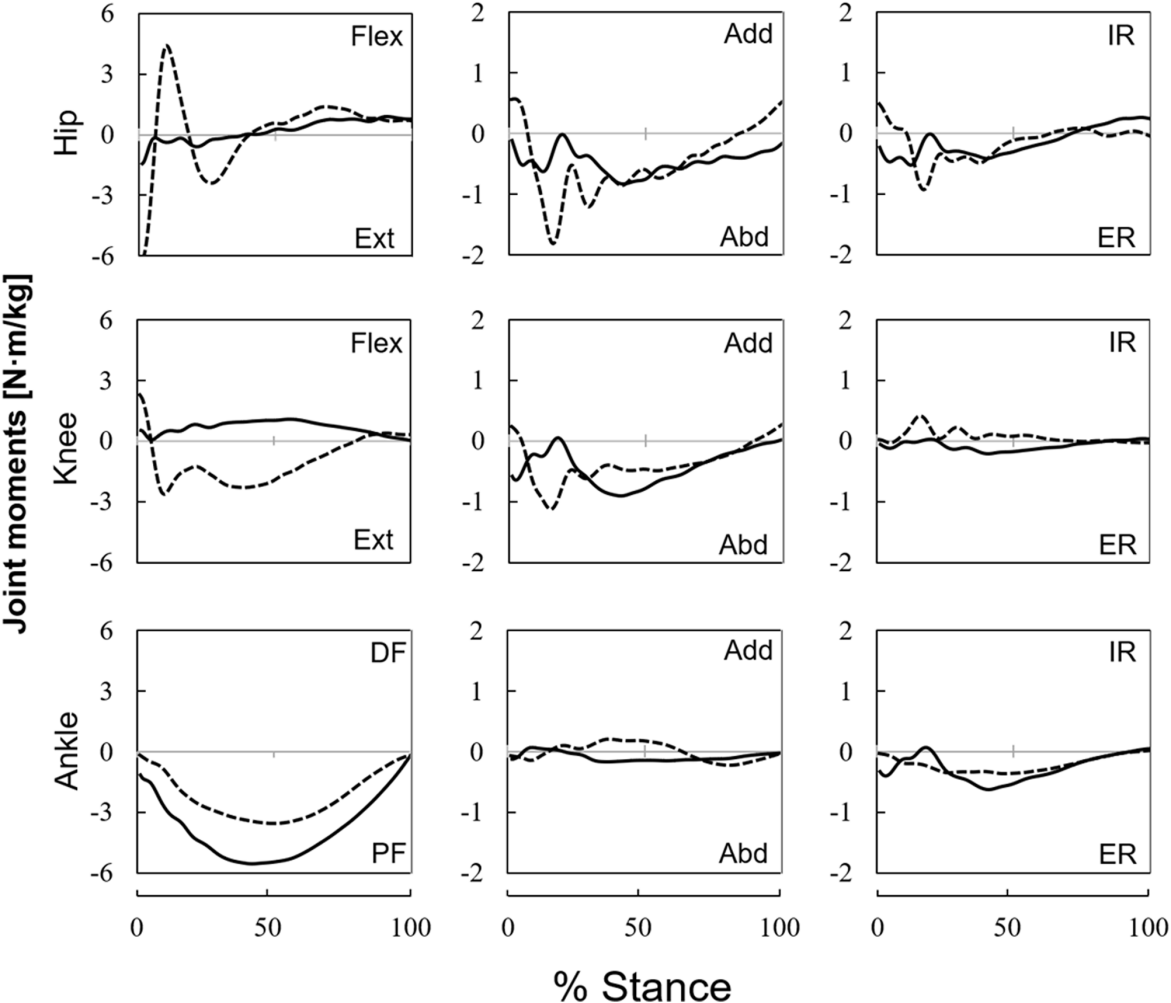
**Average time-course profiles of joint moments.** PST represented by a solid lines and INT represented by dashed lines. All moments were normalized to participant body mass and stance time for each step. Positive values indicate flexion, dorsiflexion, adduction and internal rotation moments. Flex/Ext, flexion/extension; DF/PF, dorsiflexion/plantar flexion; Add/Abd, adduction/abduction; IR/ER, internal/external rotational moments.

The average sprint velocity of all participants was 5.84±0.74 m/s. The braking GRF impulse of PST and INT were 0.10±0.03 Ns/BW and 0.21±0.05 Ns/BW, respectively. The propulsive GRF impulse of PST and INT were 0.22±0.05 Ns/BW and 0.21±0.02 Ns/BW, respectively (see Fig S1 and Table S1). The average variation velocity during PST and INT stance phase were 0.12±0.07 m/s and −0.004±0.061 m/s, respectively. In other words, participants accelerated and slightly decelerated during PST and INT stance phase, indicating that joint moments reported in the present study would be of nearly maximum speed sprinting. Fig. 1 showed the hip, knee and ankle joint-moment profiles normalized to the stance phase. Overall, the joint-moment patterns differed visibly between the legs, except for the plantar flexion moment.

All peak flexion and extension moments in the PST were significantly lower than those in the INT, except for the peak plantar flexion moment (Table 1). In the frontal plane, most of the peak adduction and abduction moments in the PST were significantly lower than those in the INT (Table 1). However, there were no statistical differences in the peak knee and ankle abduction moments between the legs. Although the peak internal rotation moments of hip and knee were significantly lower in PST than INT, peak internal rotation moments of the ankle were significantly greater in PST than INT. On the other hand, there were no significant differences in the peak external rotation moments of the hip, knee and ankle between PST and INT (Table 1).

**Table 1.**
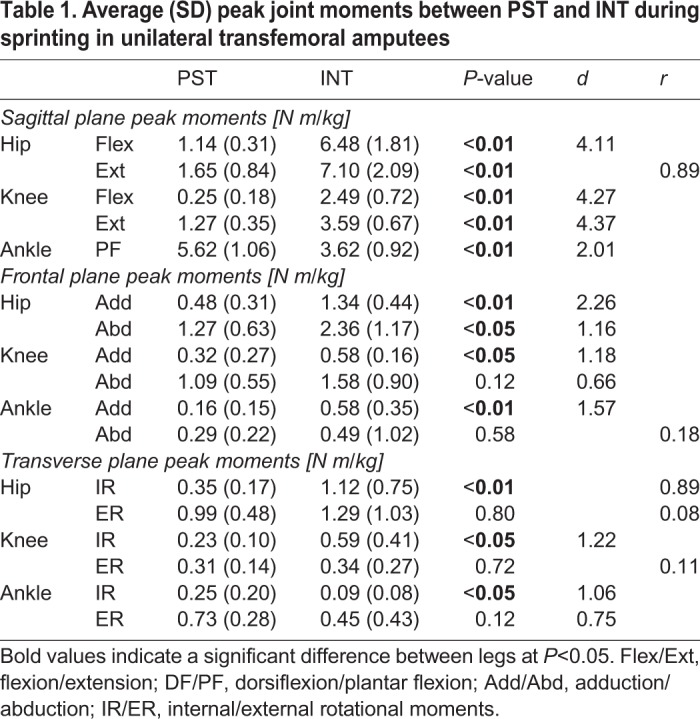
**Average (SD) peak joint moments between PST and INT during sprinting in unilateral transfemoral amputees**

## DISCUSSION

The purpose of this study was to investigate joint moments during sprinting in unilateral transfemoral amputees wearing RSPs. As expected, most of the peak hip and knee joint moments of the PST were significantly lower than those of the INT (Table 1). However, the ankle plantar flexion and internal rotation moments of the PST were significantly greater than those of the INT (Table 1). These results contrast with our initial hypothesis that the joint moments of the PST would be smaller than those of the INT in unilateral transfemoral amputees during sprinting.

### Sagittal plane

As shown in Table 1, the peak hip joint flexion and extension moments in the PST were significantly lower than those of the INT. These results agree with a past finding that unilateral transtibial amputees exhibit lower joint moment magnitudes in the prosthetic leg than in the intact and control legs throughout stance during sub-maximal running (Baum et al., 2018). Moreover, according to another previous study, 10-week training for hip strength enables transfemoral amputees to regain running capacity (Nolan, 2012). Hence, the present study and past findings suggest that the hip joint may play a major role in the process by which unilateral transfemoral amputees regain the ability to run.

It is worth noting that, in our study, the knee joint in the INT was found to generate an extension moment in the stance phase, but that in the PST exhibited a flexion moment throughout the stance phase (Fig. 1). These results were not observed for unilateral transtibial amputees during running (Baum et al., 2018; Buckley, 2000), but observed for unilateral transfemoral amputees during walking at a self-selected speed (Kaufman et al., 2012; Segal et al., 2006). In the latter studies, to prevent knee buckling, the unilateral transfemoral amputees overextended their prosthetic knee joints to maintain a GRF vector at an anterior position rather than at the prosthetic knee joint center. Although the external knee extension moment was calculated in those studies (Kaufman et al., 2012; Segal et al., 2006), in our study, the internal joint moments were calculated. As our participants used hydraulic and passive prosthetic knee joints, the internal knee flexion moment was produced in the PST as a consequence of the external knee extension moment. Therefore, the current results suggest that the internal knee flexion moment during the stance phase in the PST is a compensatory strategy to prevent knee buckling.

**Fig. 2.**
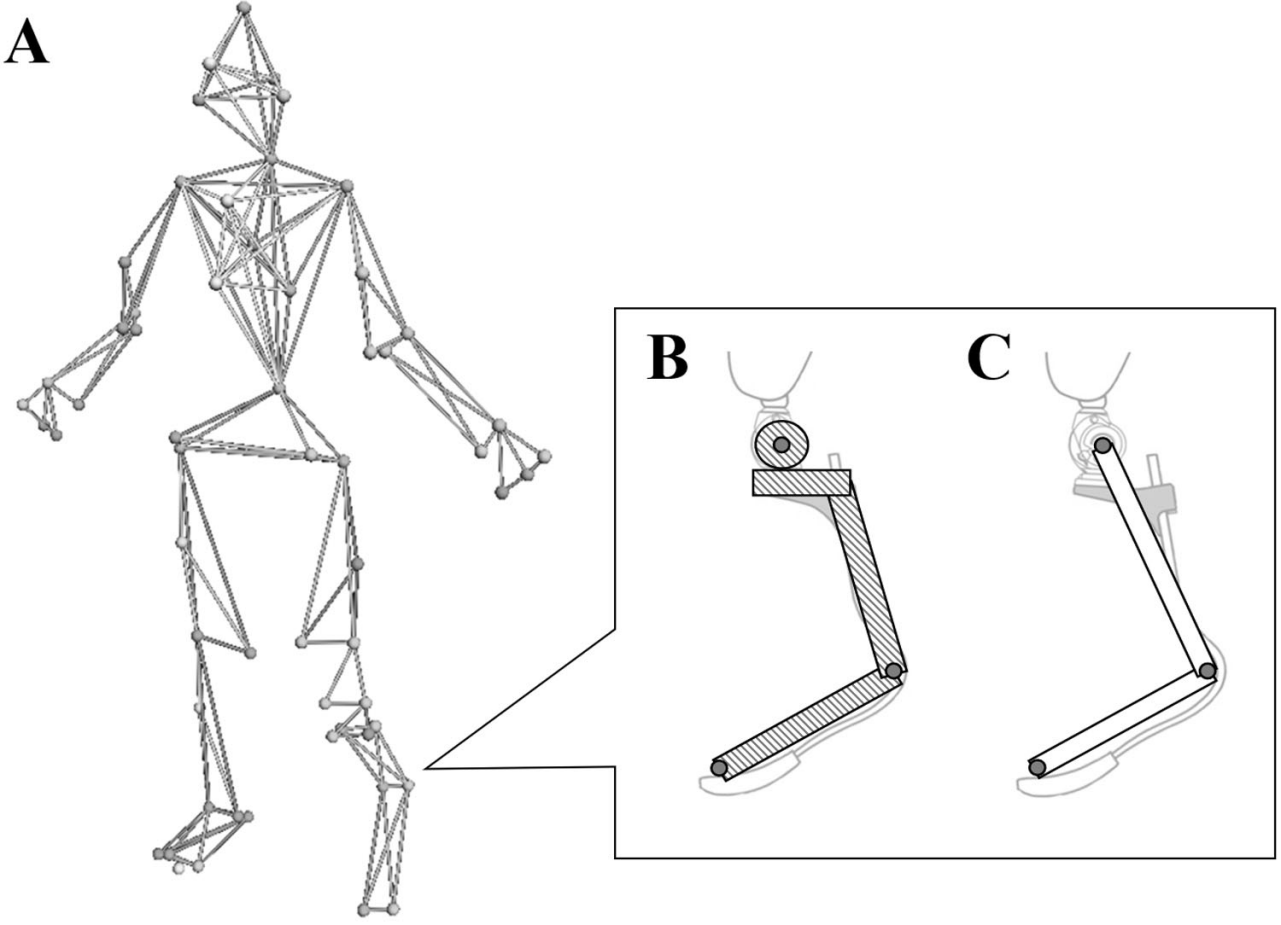
(A) Marker placement on body. (B) Model used to calculate inertial properties of each prosthetic component. (C) Definition of ankle joint composed of shank and foot segments.

The PST plantar flexion moment was significantly greater than that in the INT (Table 1). This result agrees with a previous study, which found that transtibial amputees wearing RSPs exhibit greater plantar flexion moment in the PST than in the INT during running at 2.5–3.5 m/s (Baum et al., 2018). Plantar flexion moment during stance is mainly influenced by GRF and corresponding moment arm between the line of action of GRF and the ankle joint axis. Grabowski et al. (2010) demonstrated that GRF in PST during running in unilateral transtibial amputees was smaller than that in INT regardless of increasing speed. Further, Makimoto et al. (2017) also found that unilateral transfemoral amputees during sprinting exhibit significantly smaller GRFs in the PST than in the INT. On the other hand, in the present study, the PST ‘ankle’ joint center was defined as the acute point of RSP, where PST would have relatively longer moment arm of the GRF than INT (Fig. 2). Hence, it is a reasonable assumption that the greater PST peak plantar flexion moment than INT in the present study can be attributed to the longer moment arm of the GRF, rather than to smaller GRFs.

### Frontal plane

The time-course profiles of the hip and knee joint moments in the INT obtained in this study were similar to those of able-bodied subjects (Gehring, 2014). According to Gehring (2014), the hip joint in able-bodied subjects was typically in an adducted position during running, as the foot must be placed almost directly beneath the center of mass of the body in the frontal plane. Further, a previous study demonstrated that unilateral transfemoral amputees during maximal sprinting generated GRF in medial direction on both limbs during stance phase (Makimoto et al., 2017). Consequently, the hip and knee joints generate internal abduction moments. In the present study, most of the peak joint moments in the PST were found to be significantly lower than those in the INT (Table 1). However, there were no statistical differences in the peak knee and ankle abduction moments of the different legs (Table 1). Although the joint moments of unilateral transtibial amputees during running were investigated in previous studies, only the joint kinetics in the sagittal plane were reported (Brüggemann et al., 2008; Buckley, 2000; Sanderson and Martin, 1996). As unilateral transfemoral amputees produce asymmetric joint moments in the frontal plane between the legs, knowledge of the joint kinetics in this plane may be necessary for future design and development of RSPs.

### Transverse plane

Although the hip and knee peak internal rotation moments in the PST were significantly lower than those in the INT, the ankle internal moment in the PST was significantly greater than that in the INT (Table 1). Previous studies demonstrated unilateral transfemoral amputation reduced muscle mass and strength around the hip joint on the amputated side (Burger et al., 1996; Gottschalk and Stills, 1994). Additionally, in this study, the upper and lower parts of the prosthetic knee were fixed to a carbon socket, and to the adapter parts and RSP, respectively. As transfemoral amputees cannot control prosthetic components directly, they may be compelled to generate lower internal rotation moments in the PST than in the INT in order to ease the control demands of the PST. However, the ankle internal rotation moment in the PST was found to be significantly greater than that in the INT (Table 1). As mentioned above, these results indicate that it may not be possible to neglect the joint moments in the transverse plane, and that manufacturers should take these moments into consideration during RSP development.

### Limitations

There are certain considerations that must be acknowledged when interpreting the results of the current study. First, the length of the residual limb was not considered to compute the joint moments. Although we recruited ten unilateral transfemoral amputees, we did not measure the residual limb length accurately. The difference in residual limb length could affects thigh mass and mass distribution. Moment of inertia is defined as the product of the mass of the element and the square of the shortest distance from the axis to the element. Therefore, residual limb length may be taken into account for joint moment calculation in unilateral transfemoral amputees. Indeed, a previous study reported that the residual limb length could affect the hip joint moment during sit to stand and stand to sit in transfemoral amputees (Highsmith et al., 2016). Secondly, in the present study, we defined the ‘ankle’ joint in the PST as the most acute point of the RSP. Note that Baum et al. (2018) has stated that the marker position of the ‘ankle’ joint affects the value of the joint moment. In addition, the participants of this study all used the same RSP type (Sprinter 1E90 and 3S80) during the experiment. According to previous studies, running mechanics involving RSPs may also be influenced by the RSP type (Lechler and Lilja, 2008; Nolan, 2008). Finally, although the current experiment was performed at nearly maximum constant speed sprinting, the participants tended to show slight acceleration during PST stance (see Table S1). Since the mechanics of maximum constant speed sprinting are different from those in the acceleration phase (Willwacher et al., 2016), our results may not necessarily be applicable to actual sprinting in a competitive setting. Therefore, caution is required regarding interpretation and generalization of the findings of the present study.

The purpose of this study was to investigate internal joint moments during sprinting in unilateral transfemoral amputees wearing RSPs. The PST joint moments were generally lower than those of the INT, except for the ankle plantar flexion and internal rotation moments. The results of the present study suggest that unilateral transfemoral amputees perform asymmetric joint moment adaptations to compensate for replacement of the biological leg with a passive prosthetic knee joint and RSP.

## MATERIALS AND METHODS

### Participants

Ten sprinters with unilateral transfemoral amputation participated in this study (six males and four females; age, 32.4±11.0 years; height, 1.62±0.09 m; mass, 57.1±9.0 kg; 100-m personal records, 16.9±2.4 s; mean±s.d.). All participants used the same type of prosthetic hydraulic knee joint (3S80, Ottobock, Duderstadt, Germany) and RSP (category 2 or 3 1E90 Sprinter, Ottobock, Duderstadt, Germany) with rubber soles. All participants used their RSPs for training and competition for more than 9 months. The experimental protocol was approved by the local institutional review board, and written informed consent was obtained from all participants and the guardian of one participant before the experiment.

### Experimental procedures

Before the experiment, the participants performed warm-up exercises to familiarize themselves with the experimental environment. Next, the participants performed maximum sprinting over an indoor 40-m straight runway, where seven force platforms (60 cm×40 cm, five BP400600-1000 and two BP400600-2000, AMTI, Watertown, MA, USA) and optical motion capture cameras (VICON MX system, Oxford Metrics Ltd, UK) were placed approximately 22 m from the starting line. Successful trials were defined as those in which the participants stepped within the force platform boundaries. In cases in which the step overlapped two force platforms, the data of those platforms were cumulated. In the experiment, four successful steps of each leg were collected. All participants were given adequate rest between trials.

### Data collection and analysis

Prior to beginning the experiment, a total of 59 retro-reflective markers were attached to the bony landmarks, prosthetic knee joint and RSP (Fig. 2A). All markers on the body (excluding the prosthetic segments) were placed based on the Helen Hayes marker set (van Sint Jan, 2007). We collected GRFs using force platforms at 2000 Hz and three-dimensional marker positions using the VICON MX motion capture system with 20 cameras at 200 Hz. The raw GRFs and three-dimensional marker positions were filtered using a fourth-order zero-lag low-pass Butterworth filter with cut-off frequencies of 75 (Hunter et al., 2004) and 20 Hz (Kuitunen et al., 2002), respectively. Then, the GRF data was downsampled to 200 Hz to correspond to the kinematic data. The forward velocity during sprinting was calculated as the forward displacement of the sacral divided by the time required for the sacral marker to pass over the seven force platforms (Makimoto et al., 2017; Sano et al., 2017). Further, we calculated the braking and propulsive GRF impulse of PST and INT to confirm whether participants accelerated/decelerated or not. The variation velocity during PST and INT stance time were calculated by law of conservation of momentum, respectively.

Based on a previous study (Buckley, 2000), the prosthetic ‘ankle’ joint was defined by the markers on the most acute point on the prosthesis curvature attached to the midpoint between the RSP medial and lateral edges. The coordinate system embedded in the prosthetic shank segment was defined by the following four landmarks: the prosthetic knee joint (medial/lateral) and prosthetic ‘ankle’ joint (medial/lateral). The coordinate system embedded in the prosthetic foot was defined by the following four landmarks: the prosthetic ‘ankle’ joint (medial/lateral) and toe (medial/lateral). The masses and moments of inertia for each segment in the intact and residual legs were estimated using the method proposed by Hanavan (1964). The moments of inertia of the prosthetic shank and foot segments were estimated by assuming that those parts had homogeneous solid cylinder and thin plate forms, respectively (Fig. 2B,C). The mass of each component was taken to be the officially launched value (Ottobock). The joint moments were calculated through an inverse dynamics method and are described in the proximal segmental coordinate system by using Visual 3D software (C-Motion, Germantown, MD, USA). The joint moments of each participant were normalized according to their body mass.

### Statistical analysis

First, we performed a Kolmogorov–Smirnov test to confirm the data distribution. If normality was observed, paired *t*-tests were conducted to compare the hip, knee and ankle joint variables (peak moments) of the INT and PST. If normality was violated, a Wilcoxon signed-rank test was conducted. The effect size (ES) was also calculated for the dependent *t*-test as *d*, which is a Cohen's d-like normalized effect size measure, and for the Wilcoxon signed rank sum test using ES correlation indicators (*r*). The ES (*d*) was calculated as the ratio of the mean of the difference vector and the standard deviation of the distance vector. In this study, ES values of <0.5, 0.5−0.8, and >0.8 were regarded as small, medium and large differences, respectively. SPSS for Windows Version 22 (SPSS Inc. Chicago, IL, USA) was used for all statistical analyses. Statistical significance was set to *P*<0.05 and ES>0.8.

## Supplementary Material

Supplementary information
