# Supplementary material for: Joint moments during sprinting in unilateral transfemoral amputees wearing running-specific prostheses
Source: Biol Open. 2019 Jan 23;8(2):bio039206. doi: 10.1242/bio.039206 (PMC6398468; doi:10.1242/bio.039206)
Supplement: Supplementary information [file biolopen-8-039206-s1.pdf]

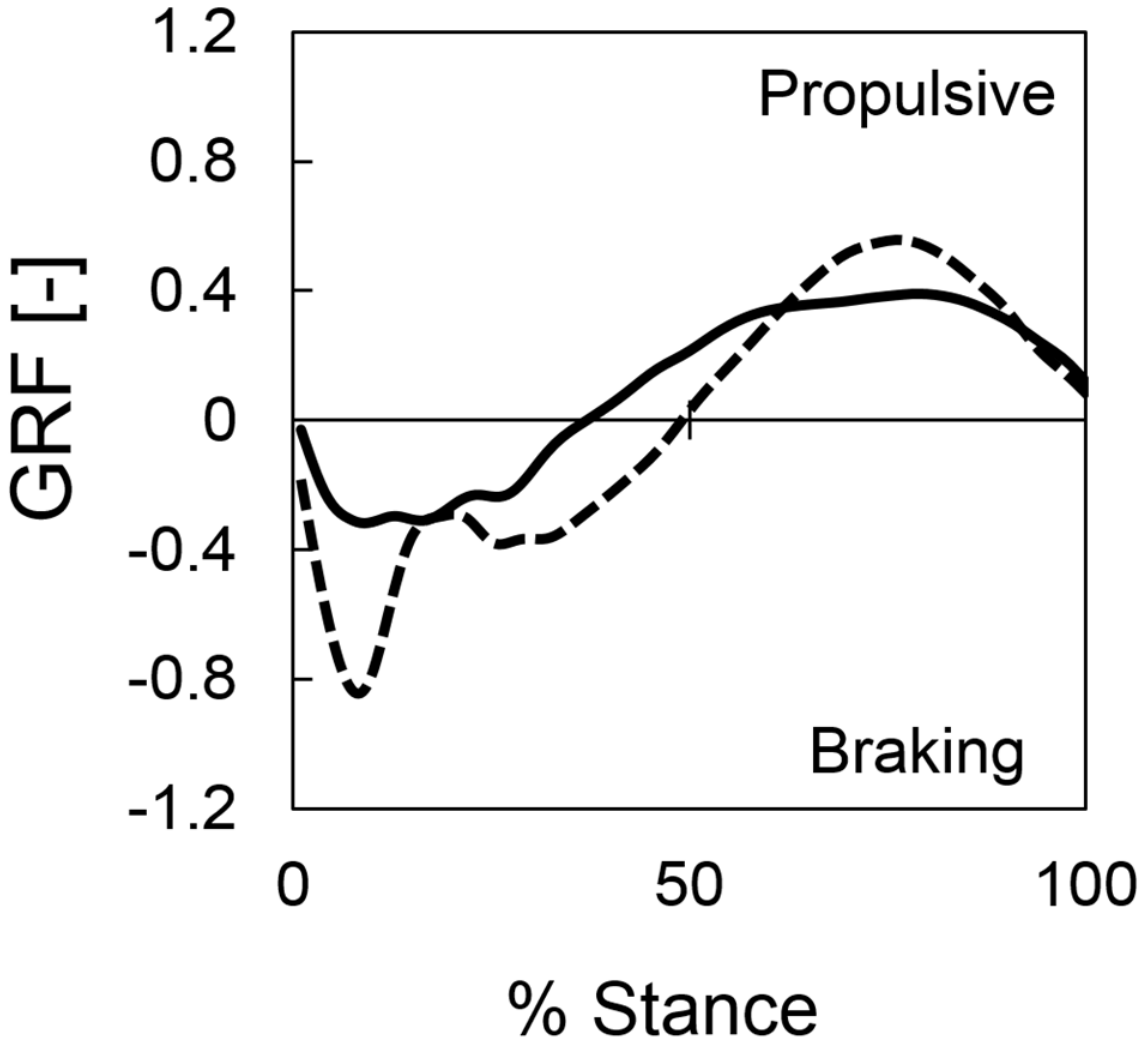

Figure S1. Average time-course profiles of braking and propulsive GRF in PST (black line) and INT (dotted line). GRF were normalized to participant body weight and stance time for each step. Negative and positive values indicate braking and propulsive GRF, respectively.

Biology Open • Supplementary information

Table S1. Average (SD) impulse of GRF between PST and INT during sprinting in unilateral transfemoral amputees.

	PST	INT
Net GRF impulse [Ns/BW]	0.12 (0.07)	0.00 (0.06)
Braking GRF impulse [Ns/BW]	0.10 (0.03)	0.21 (0.05)
Propulsive GRF impulse [Ns/BW]	0.22 (0.05)	0.21 (0.02)
